# Metformin and its potential influence on cell fate decision between apoptosis and senescence in cancer, with a special emphasis on glioblastoma

**DOI:** 10.3389/fonc.2024.1455492

**Published:** 2024-08-29

**Authors:** Melika Hajimohammadebrahim-Ketabforoush, Alireza Zali, Mohammadreza Shahmohammadi, Amir Ali Hamidieh

**Affiliations:** ^1^ Student Research Committee, Department of Clinical Nutrition and Dietetics, Faculty of Nutrition Sciences and Food Technology, National Nutrition and Food Technology Research Institute, Shahid Beheshti University of Medical Sciences, Tehran, Iran; ^2^ Functional Neurosurgery Research Center, Shohada Tajrish Comprehensive Neurosurgical Center of Excellence, Shahid Beheshti University of Medical Sciences, Tehran, Iran; ^3^ Pediatric Cell and Gene Therapy Research Center, Gene, Cell & Tissue Research Institute, Tehran University of Medical Sciences, Tehran, Iran

**Keywords:** metformin, GBM, apoptosis, senescence, calorie restriction, AMPK, mTOR, P53

## Abstract

Despite reaching enormous achievements in therapeutic approaches worldwide, GBM still remains the most incurable malignancy among various cancers. It emphasizes the necessity of adjuvant therapies from the perspectives of both patients and healthcare providers. Therefore, most emerging studies have focused on various complementary and adjuvant therapies. Among them, metabolic therapy has received special attention, and metformin has been considered as a treatment in various types of cancer, including GBM. It is clearly evident that reaching efficient approaches without a comprehensive evaluation of the key mechanisms is not possible. Among the studied mechanisms, one of the more challenging ones is the effect of metformin on apoptosis and senescence. Moreover, metformin is well known as an insulin sensitizer. However, if insulin signaling is facilitated in the tumor microenvironment, it may result in tumor growth. Therefore, to partially resolve some paradoxical issues, we conducted a narrative review of related studies to address the following questions as comprehensively as possible: 1) Does the improvement of cellular insulin function resulting from metformin have detrimental or beneficial effects on GBM cells? 2) If these effects are detrimental to GBM cells, which is more important: apoptosis or senescence? 3) What determines the cellular decision between apoptosis and senescence?

## 1 Introduction

Glioblastoma multiforme (GBM) is known as the most devastating and incurable primary brain tumor. Patients with GBM experience a wide range of side effects and discomforts, resulting in an average life expectancy of approximately 14 months (1–4). Therefore, more of them are seeking additional remedies. Current treatments for GBM include surgery to remove as much of the tumor as possible, followed by radiation and temozolomide (TMZ) (5–7). Moreover, the use of nitrosourea-based regimens, especially lomustine (oral form) and carmustine (infusion form), has been evidenced as efficient adjuvant therapy in GBM (8–10). However, existing therapeutic strategies are still controversial, and it has remained an incurable disease in health services so far (4, 11). Therefore, even the smallest effort to find new insights into GBM therapy may be extremely valuable. According to it, complementary medicines and diets have already been imported and studied in the literature (12–17). Among these studies, the results are still very controversial. For example, recent studies have discussed how a ketogenic diet could improve GBM prognosis by reducing tumor size, tumor progression, and enhancing survival and quality of life. These impacts may be attributed to the anti-tumor properties of ketone bodies (18, 19), However, studies from a mechanistic perspective suggest that increased activation of fatty acid synthase has progressive effects on GBM cells (20, 21). Studies on the benefits of statin drugs in GBM patients have confirmed these results (4). On the other hand, blood glucose and serum insulin levels are significant metabolic culprits in the extensive body of cancer studies. An increasing number of recent studies have confirmed the benefits of metformin for cancer patients, including those with GBM (4, 22–27). However, it is still not well known whether these benefits truly result from reducing glucose and systemic insulin, or if other mechanisms are involved. It is noteworthy that metformin is recognized as an insulin-sensitizing drug that enhances insulin function at the cellular level, leading to reduced systemic insulin levels. With careful focus on literature, it is well established that metformin exerts anti-tumor effects on various cancer types through various possible routes, including cancer metabolism, epigenetics, cell cycle arrest, cancer invasion, migration, and metastasis, cell death, senescence, cancer stem cells, cancer immunity, and gut microbes, as demonstrated in *in vitro* and *in vivo* studies (28–41). The activation of adenosine monophosphate-activated protein kinase (AMPK), phosphorylation, and activation of P53, leading to the apoptotic state, is the most frequently mentioned mechanism of metformin function in many studies on various types of cancer (23, 42, 43) and GBM (44–50). In this regard, some studies have discussed both apoptosis and senescence as forms of cell death. However, it is well known that senescence is a state in which a cell no longer divides but has not yet undergone cell death. Moreover, unlike apoptosis, senescence is associated with the senescence-associated secretory phenotype (SASP) and resistance to apoptosis (51–54). Among the studies, most have focused on insulin, metformin, and apoptosis in GBM, a recent high-quality study suggested that metformin and simvastatin alone, but especially their combination, result in decreased cell proliferation and VEGF, increased apoptosis, and senescence in GBM cells (4). However, because senescence is associated with SASP and could induce immunosenescence, recent studies have shown that metformin can delay aging by reducing cellular senescence (29, 55, 56). On the other hand, taken together, to the best of our knowledge, insulin has a growing effect on cancer cells (57–59), therefore, the study mentioned above (4) may raise questions about how metformin’s improvement of insulin function at the cellular level could lead to anti-tumor effects on GBM cells. Among these enormous controversies, it seems that finding the exact mechanisms could partially help to solve these issues and to use supplementary drugs and diets to enhance the recovery of GBM patients. The present study aimed to conduct a narrative review of related studies to address the following questions as comprehensively as possible: 1) Does the improvement of cellular insulin function resulting from metformin have detrimental or beneficial effects on GBM cells? 2) If these effects are detrimental to GBM cells, which is more important: apoptosis or senescence? 3) What determines the cellular decision between apoptosis and senescence?

## 2 The studied mechanisms of the antitumor effects of metformin, especially in GBM

**Figure 1:**
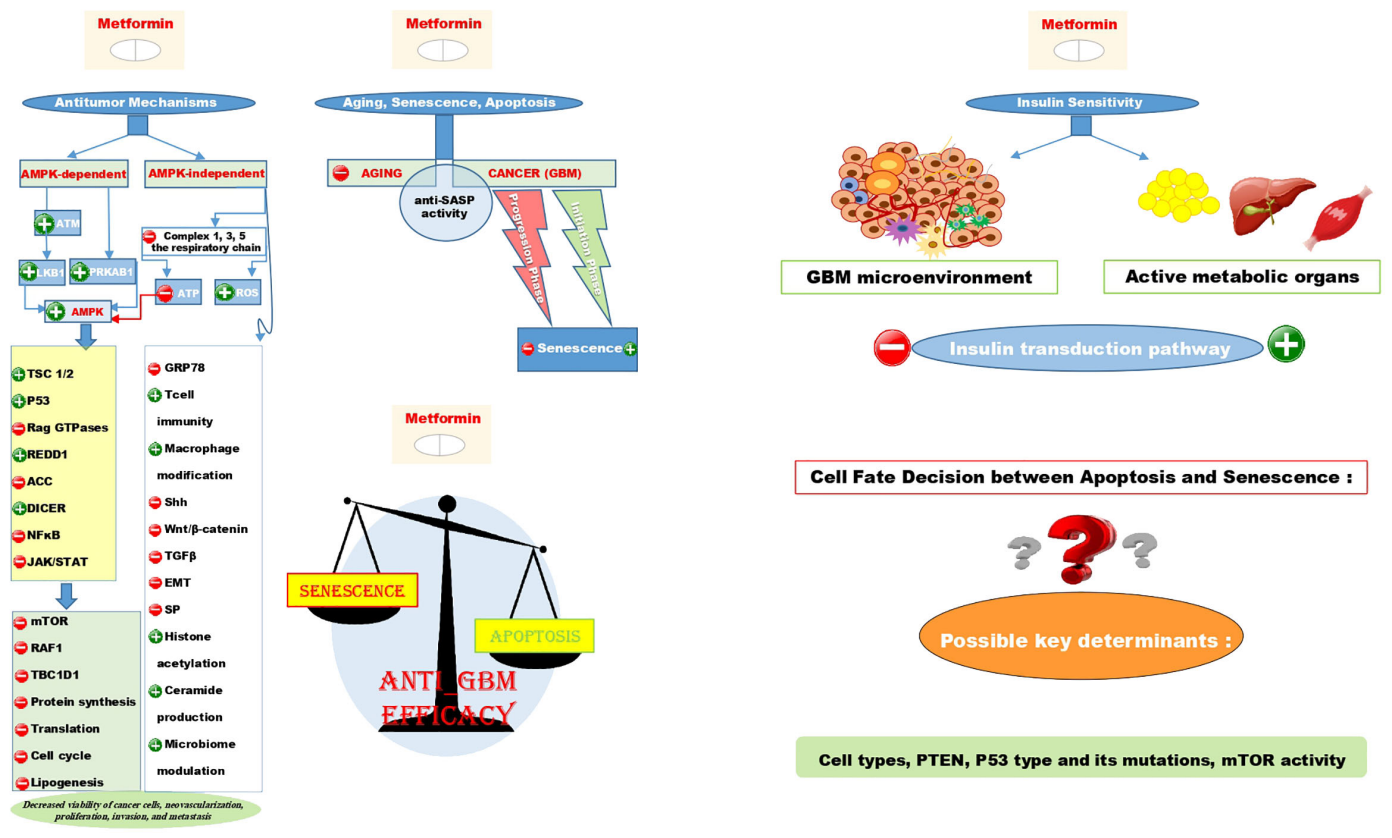
Illustrates the AMPK-dependent and AMPK-independent antitumor mechanisms of metformin using + signs for induction and - signs for repression on the left side of the figure. It also shows that the anti-SASP activity contributes to both the anti-aging and anti-cancer effects of metformin. Interestingly, metformin can induce senescence in the initiation phase of GBM while repressing it in the progression phase. The scale schematic suggests that the primary anti-tumor effect of metformin is linked to apoptosis rather than senescence, with cell types, PTEN, P53 type and mutations, and mTOR activity as key factors in determining cell fate decision between these two situations. Additionally, despite metformin being an insulin sensitizer metabolically, it could induce insulin resistance in the GBM microenvironment.

Mechanistically, studies have discussed multiple major aspects of the antitumor efficacy of metformin. These aspects are almost always related to systemic, cell-autonomous pathways, which can be AMPK-dependent or independent (60–66). Although there are innumerable underlying mechanisms, and their comprehensive explanations may not be entirely satisfying, we have made our best effort to review the most important ones as thoroughly as possible. The anticancer mechanisms of action for metformin, along with the possible responses to the rest of our research queries, have been summarized in a graphical abstract in **Figure 1**.

### 2.1 AMPK-dependent mechanisms

AMPK-dependent pathways are most frequently mentioned, identifying AMPK as a master regulator in this regard. Genome-wide association studies have confirmed that metformin can induce the Ataxia-telangiectasia mutated kinase (ATM) in cancer cell lines (67–71). The activated ATM phosphorylates Liver kinase B1 (LKB1) at Thr366, and subsequently, the activated LKB1 phosphorylates and activates AMPK at Thr172 (60). Notably, some studies have suggested that metformin also significantly increases the concentration of the regulatory subunit of AMPK (PRKAB1), which interacts with the catalytic subunit (61). Ultimately, metformin activates AMPK, exerting anti-tumorigenic properties in cancers by phosphorylating and activating two crucial tumor suppressors, TSC 1/2 and P53 (28, 65, 72, 73). The activation of TSC 1/2 could inhibit the mammalian target of rapamycin (mTOR) signaling, thereby downregulating proliferation-related proteins such as RAF1 and TBC1D1, which in turn inhibits protein synthesis, translation, and cell cycle progression (61). Furthermore, metformin inhibits Rag GTPases and activates REDD1 (regulated in development and DNA damage responses 1), a negative regulator of mTOR, resulting in the blockage of mTOR signaling. These results indicate the inhibition of both protein synthesis and gluconeogenesis (61, 65, 74). At the same time, activation of AMPK by metformin puts the body into a state of starvation and catabolism, leading to the repression of protein synthesis and lipogenesis. This repression occurs through the inhibition of Acetyl CoA Carboxylase (ACC) and fatty acid synthetase (FASN), ultimately resulting in decreased viability of cancer cells, neovascularization, proliferation, invasion, and metastasis in both *in vitro* and *in vivo* studies (75–79).

#### 2.1.1 Mitochondrial respiratory chain complexes

On the other hand, mitochondria have always been the best therapeutic target in cancer because mitochondrial activity and biogenesis are vital for tumor cell activity. Interestingly, studies have suggested that metformin can enter cancer cells via the organic cationic transporter (OCT) and directly affect mitochondria (66, 80). In this way, metformin inhibits complex 1 of the respiratory chain, alongside decreased protein subunit complex 1 and fifth components of complex 3. Subsequently, increased reactive oxygen species (ROS) resulting from respiratory chain disruption leads to DNA damage and apoptosis (61, 81, 82). The inhibitory effects of metformin on the mitochondrial respiratory chain led to decreased ATP production, causing an energetic stress state and activating AMPK and its subsequent hierarchy as mentioned in the previous section.

#### 2.1.2 Cell cycle

Most authors believe that the antitumor effects of metformin are primarily mediated by a disruption in the cell cycle, which is caused by the activation of P53 induced by AMPK. Exposure to metformin further activated P53, leading to apoptosis and autophagy. It induced cell cycle arrest at the G0/G1 and G2/M phases by activating P21, P27, Bax, cyclin E, and decreasing cyclin D1 consequently downregulating cyclin-dependent kinases such as CDK1,CDK2,CDK4, and CDK6 in various cancer cell lines (83–86). Moreover, it has been suggested that a decrease in ATP production in mitochondria can lead to a pro-apoptotic (Bax)/anti-apoptotic (Bcl2) imbalance, followed by the entry of Bax into the mitochondria and the release of cytochrome C, which is a signal for cell death (87, 88).

#### 2.1.3 MicroRNAs

The AMPK-dependent antitumor effects of metformin extend beyond this. Studies have shown that AMPK is a positive regulator of DICER, which in turn leads to alterations and modifications in various subpopulations of microRNAs. Both basic and clinical studies have shown the importance of inhibiting microRNA expression in cancer pathogenesis (89–92). DICER, a helicase with an RNase motif, can selectively activate the RNA-induced silencing complex (RISC), resulting in the inhibition of translation or fragmentation of the oncogenic target mRNA. This novel discusses the antitumor mechanism recently attributed to metformin in studies.

#### 2.1.4 Inflammation

AMPK can also disrupt inflammatory pathways, thereby exerting metformin-dependent antitumor effects. In this way, by blocking the NFκB signaling, which is an important factor for tumor progression in inflammation-related cancers, and by decreasing TNF-α and some inflammatory cytokines such as IL-6, tumor progression can be alleviated (93–95). Furthermore, evidence has shown that metformin can stimulate CD8 cells and induce T-cell immunity, thereby attenuating cancer and inhibiting cancer occurrence in various cancer studies, respectively (96–98). Interestingly, metformin can suppress inflammation through NFκB signaling inhibition mediated by AMPK, or through AMPK-independent pathways as suggested in studies (65, 99, 100). This suppression can lead to a transition of the tumor-associated macrophage M2 phenotype to the M1 phenotype, causing macrophages and their secretions to attack the tumor itself rather than promoting its progression. Recent studies have mentioned these mechanisms, along with those related to the regulation of DICER, as potential anticancer stem cell (Anti-CSC) mechanisms (65).

### 2.2 Possible AMPK-independent mechanisms

Some other metformin anti-CSC mechanisms, which are completely unrelated to the AMPK signaling, involve the direct inhibition of self-renewal and metastatic pathways, including Sonic Hedgehog (Shh), Wnt/β-catenin, and TGFβ signaling. Moreover, metformin directly inhibited epithelial-to-mesenchymal transition (EMT), a dedifferentiation pathway that gives rise to a tendency for tumorigenesis and invasiveness (101–103). Another suggested mechanism is related to the unfolded protein response (UPR). When proteins are misfolded pathologically or unfolded, there are three receptors on the endoplasmic reticulum (ER) membrane that sense them and dislocate from the membrane, triggering a signal transduction for protein degradation and apoptosis. It is worth noting that metformin has been suggested as a downregulator of GRP78, a heat shock protein that regulates UPR. This can lead to an increase in unfolded protein levels in the ER lumen, causing ER stress and inducing cell death (61). It is interesting that even in this mechanism, some studies found a clue related to AMPK and attributed it to the mechanism. In this regard, metformin induces CHOP-related apoptosis in some cancer cell lines, and the regulation of certain microRNA expressions is also involved in this effect of metformin (61, 87, 104–106). Some discussions have focused on the JAK/STAT pathway, with studies indicating that STAT signaling is abnormally activated in most solid tumors and is associated with increased proliferation and invasion properties. Recent evidence has shown an AMPK-dependent effect of metformin that leads to limited STAT signaling in cancers (66). As we have reviewed so far, the antitumor effects of metformin are often directly or indirectly mediated by AMPK. However, some of the mentioned mechanisms appear to be free from any clues of the AMPK. For instance, previous studies have suggested that metformin may induce epigenetic modifications. Some authors have reported that metformin can inhibit members of the transcription factor family SP, especially SP1 (107, 108). This inhibition has been linked to the downregulation of several pro-oncogenic genes, including Bcl2, survivin, cyclin D1, VEGF, and FASN, in various *in vitro* and *in vivo* cancer studies. Furthermore, metformin-mediated SP1 degradation led to the downregulation of the oncogenic isoform of pyruvate kinase M2. Overall, inhibition and degradation of SP1 have been reported to be associated with the control of most SP1- and metabolic pathways-related cancers (61). Moreover, the inhibition of the tricarboxylic acid cycle, leading to the accumulation and forwarding of acetyl CoA for histone and non-histone acetylation, is reported as another epigenetic antitumor mechanism of metformin (109). On the other hand, metformin disrupts sphingolipid metabolism, thereby interfering with onco-promoter signaling and increasing ceramide production, a pro-apoptotic agent (110). It has also been suggested that metformin directly disrupts the integrity of the mitochondrial membrane and induces apoptosis in cancer cell lines by disrupting calcium flow (111). Recent and novel evidence has discussed the gut microbiota-related antitumor effects of metformin in both *in vitro* and *in vivo* studies. In these studies, metformin was found to modulate the gut microbiome, leading to decreased carcinogenesis and tumor progression, particularly in colorectal cancers (28, 112).

### 2.3 Focusing on GBM

To the best of our knowledge, the underlying antitumor mechanisms of metformin that have been mentioned so far are reported as the most frequent and important in various cancers, including breast (22), pancreas (78), ovary (113), lung (114), liver (77), esophagus (83), gastric (33), colorectal (115), thyroid (116), endometrial (85), prostate (106), melanoma (117), leukemia (118), and myeloma (86). However, there are still limited studies related to brain tumors. Some studies have shown similar mechanisms to those mentioned in previous sections also in cases of glioma and glioblastoma. For instance, Moretti et al. (119) evaluated the effects of metformin on two GBM cell lines that were stimulated with lipopolysaccharide (LPS) as an agonist for the TLR4 pathway. Metformin, when used alongside TMZ in both GBM cell lines, leads to the disruption of mitochondrial respiration, resulting in oxidative stress. In the mentioned study, decreased cell viability, increased apoptosis due to ER stress, and downregulation of BCL2 were observed after these treatments. Moreover, they have demonstrated through in silico analysis of TCGA-GBM-RNASeq that GPM-GBM cases with an activated TLR4 pathway may benefit from metformin treatment, but the simultaneous upregulation of CXCL8/IL8 may require a combination therapy with an IL8 inhibitor. Metformin combined with an antioxidant inhibitor, such as anti-SOD1, may be recommended for cases of mitochondrial-GBM. In another study, Song et al. (64) have shown that metformin inhibits the invasive properties of GBM cells by blocking the epithelial-to-mesenchymal transition-like process and consequently inhibiting the related TGF-β1 pathway. As a whole, these studies have illustrated that metformin plays an anti-cancer stem-like role in GBM cell lines via the AKT/mTOR/ZEB1 pathway. This provides evidence of metformin for further clinical investigation targeting GBM. As highlighted previously in this review, metformin can enter mitochondrial or other tissue and cell membranes, as well as cancer cells, via the OCT transporter. Furthermore, studies have shown that many transporters are identified for facilitating the entrance of metformin into cells based on the type of organs. For example, the carnitine/organic cation transporter 2 (OCTN2), plasma membrane monoamine transporter (PMAT), thiamine transporter 2, and serotonin transporter are attributed to intestinal cells through which metformin enters the cells and then shifts to the blood. On the other hand, multidrug and toxin extruder (MATE) 1 and 2 are responsible for metformin entering renal tubes and then shifting to urine (120). Although the blood-brain barrier (BBB) is tightly conserved, animal studies have shown that after a few hours of metformin administration, there is an equal concentration of metformin in brain tissue and plasma (121). This interesting fact indicates that metformin can somewhat penetrate the BBB. The exact mechanism is not clearly defined yet, but some studies suggest that four types of organic cationic transporters - OCT1, OCT2, OCT3, and PMAT, as well as plasma glycoprotein and MATE-1, are responsible for metformin penetration into the brain through the BBB (120, 122–124). Also, recent studies suggest that the CLIC1 protein, which is sensitive to metformin, accumulates in the transmembrane of GBM stem cells and can lose its function due to metformin, demonstrating the antitumoral action of metformin in the brain (125, 126). The exact effective concentration of metformin in the brain still remains to be well understood in the literature. However, some *in vitro* studies have reported antitumor effects of metformin with increasing concentrations in a dose-response manner in GBM cell lines (4, 127–130). One study showed that low metformin concentrations are related to cytostatic effects, while higher concentrations (about 10 mM) had cytotoxic effects on GBM-initiating cells (131). Another study demonstrated that metformin concentrations beyond 2.5 mM reached a plateau in terms of anti-GBM activity (127). Moreover, in terms of the response of GBM to metformin, the following theme could be worth noting. O6-methylguanine (O6-MeG)-DNA methyltransferase (MGMT) is considered a clinical biomarker in GBM based on numerous previous studies (132). The primary reason for this is the frequent occurrence of drug resistance issues in these patients. The key mechanism of the cytotoxic efficacy of TMZ, the first-line chemotherapy in GBM, is the alkylation of DNA, which subsequently hinders replication. In this process, a methyl group is added to the O^6^ N^7^ position of guanine and the N^3^ position of adenine. This methylation leads to mispairing of bases and breaks in DNA strands. MGMT, through the transfer of the methyl group, disrupts this process and hinders the anti-GBM effect of TMZ (132–134). Studies have indicated that MGMT promoter status is a crucial factor in determining TMZ sensitivity (132, 135). A methylated promoter results in MGMT gene silencing, thereby enhancing TMZ efficacy (132). However, evidence shows that 30-60% of patients with a methylated MGMT (mMGMT) promoter still exhibit TMZ resistance due to MGMT expression (132). Interestingly, one study suggested that combining metformin with TMZ and/or radiotherapy enhances the efficacy of these treatments, even in resistant GBM cells with unmethylated MGMT promoters (133). On the other side, studies have discussed that metformin alone or in combination with TMZ can significantly suppress the induction of MGMT proteins in a dose-response manner (134). Furthermore, some studies have suggested that metformin can sensitize TMZ-resistant GBM cells through the suppression of MGMT expression and more detailed mechanisms. Therefore, it seems that metformin could be a suitable adjuvant therapy in the current situation where there is no substitute for TMZ. In practice, scientific associations still believe in the standard of care based on TMZ even in resistant GBM cells with unmethylated MGMT promoter due to the indefinite predictive value of MGMT promoter status. However, a recent cohort study belonging to the *JAMA* network (136) has shown that glioma patients with mMGMT promoter had experienced longer survival than those with unmethylated MGMT promoter in terms of both progression-free survival and overall survival. Interestingly, some studies have shown that metformin use had a beneficial effect on survival in GBM patients with mMGMT promoter (137). Altogether, it seems that there is a need for more rigorous trials to evaluate these effects, considering pivotal factors such as MGMT promoter status and excluding patients with diabetes.

## 3 The effects of metformin on cellular insulin function in healthy cells compared to GBM cells

It is evident that after insulin interacts with its receptor and undergoes autophosphorylation of the insulin receptor substrate (IRS) cytoplasmic domain, it triggers a crucial signal transduction pathway mediated by signaling molecules such as Shc, Grb2, SoS, Ras, Raf-1, MEK. This ultimately activates ERK1/2, leading to cell proliferation and growth. Moreover, insulin activates PI3K and Akt, leading to enhanced cell glucose uptake, as well as promoting glycogen, lipid, and protein synthesis (138–141). In healthy cells, particularly in active metabolic organs, metformin, a well-known insulin sensitizer drug, enhances the effects of insulin on growth by accelerating the IRS signaling, leading to increased glucose uptake and reduced systemic circulating glucose and insulin. This, in turn, mediates the definition of metformin’s antitumor efficacy, due to the well-established tumorigenesis and tumor growth effects of high glucose levels and hyperinsulinemia in various cancer studies (142–144). Interestingly, evidence from studies on cancers and GBM cell lines discusses the fact that metformin could disrupt the insulin signaling pathway in cancerous cells by downregulating key signaling molecules in this pathway, such as PI3K, Akt, and ERK1/2. Therefore, this suggests that the impact of metformin as an insulin sensitizer may be reversed in cancer cell microenvironments, inhibiting the growth-promoting efficacy of insulin in this context (26, 27, 131, 145–148). One of the astonishing challenges in biology is the recognition that the effect of metformin varies greatly depending on the context or type of cell. A study illustrating potential molecular targets in glioblastoma has reported that the PI3K/AKT/mTOR pathway is the most significant target compared to other culprits even mutated P53 so that this pathway is overexpressed in 90% of GBM cases (27). Therefore, it seems that metformin has potential therapeutic effects on GBM by disrupting this pathway. On the other hand, there is an interesting controversy in some studies claiming that ERK1/2, a key factor in cell proliferation and growth activated by the insulin signaling cascade, could lead to cell death. These studies have indicated that ERK1/2 mediates cell death based on stimuli and activated cell types. The intensity, duration, and balance between pro-versus anti-apoptotic signals determine whether a cell duplicates or undergoes an apoptotic state (149–152). Although the exact mechanisms remain to be understood, some studies have suggested ERK-associated DNA damage (151) or IFNγ-induced cell death (153). Furthermore, the involvement of ERK1/2 in inhibiting survival signaling and Fas-mediated cell death are other proposed mechanisms in this context (150). This evidence suggests that Akt is downstream of ERK1/2 activation in the cytosol, so it does not phosphorylate ERK1/2. Instead, it phosphorylates and stabilizes PEA-15, acting as a confiscator of ERK1/2 in the cytosol. As a result, the translocation of ERK to the nuclei does not occur, and ELK-1-associated transcription is not activated (150, 152). Therefore, it seems that PI3K inhibitors, such as metformin in the context of cancer, that block Akt phosphorylation and activation, may conversely lead to the restoration of ERK1/2 translocation to the nucleus and cell growth effects. So it is noteworthy that further future studies examine the antitumor efficacy of metformin from the perspective of its effect on ERK1/2 and its translocation to the nucleus. In their valuable reports from both the orthotopic GBM mouse model and patient samples, Noch et al. (154) focused on the issue of PI3K inhibitors for GBM treatment. They highlighted that these inhibitors have failed due to their potential to elevate blood glucose, induce insulin receptor hyperactivity, trigger insulin feedback, and cause hyperinsulinemia. In this study, it is interesting to note that metformin could enhance the effectiveness of PI3K inhibition in the GBM microenvironment, while also reducing systemic glucose and insulin levels. Furthermore, PI3K inhibition led to increased T-cell and microglia presence in GBM samples from patients (154). Numerous other studies have even mentioned metformin as an independent and efficient inhibitor of the tumor niche-associated PI3K in various cancers, especially GBM. For example, Al Hassan et al. (127) demonstrated that metformin inhibits AKT, a key molecule in the PI3K signaling pathway. By blocking this pathway, metformin exerts its anticancer, anti-invasive, and anti-migratory effects on GBM cell lines. The study highlighted the significance of the PI3K/Akt pathway in glioblastoma by demonstrating reduced motility and EGF stimulating properties in GBM cells treated with wortmannin, a PI3K inhibitor. These observations were correlated with the effects observed after the use of metformin in their study. Another study by Lo Dico et al. (155) reported that they treated both GBM cell lines, including those susceptible and resistant to TMZ with metformin in combination with TMZ. During hypoxia, metformin in combination with TMZ, could decrease cell viability. This phenomenon was partly associated with the inhibition of the PI3K/mTOR axis. However, this effect on TMZ-resistant GBM cell line is facilitated by adding BEZ235, a PI3K/mTOR inhibitor. This study concluded that TMZ + metformin could reverse the resistance of GBM cells to treatment, and this effect was potentiated by a disruption in the PI3K/mTOR axis. Furthermore, Würth et al. (131) have also demonstrated that different doses of metformin treatment (4.9 - 9.4 mM) administered to four types of GBM cell lines for 48 hours resulted in decreased Akt phosphorylation, cell survival, and proliferation.

## 4 Dietary patterns that improve cellular insulin function and their effect on GBM

As frequently mentioned, metformin improves cellular insulin function in metabolic organs, leading to enhanced cellular glucose uptake and decreased circulating glucose and insulin. Furthermore, metformin activates AMPK, shifting the body into a calorie-restricted state. The well-known antitumor efficacy of metformin appears to mimic dietary patterns such as intermittent fasting, hypocaloric and calorie restriction, as well as exercise, both reasonably and mechanistically (156, 157). In this regard, some preclinical and clinical studies have shown promising results so far (158, 159). However, clinical studies examining the impact of intermittent fasting, hypocaloric diets, and calorie restriction on tumor characteristics and clinical outcomes in GBM patients have not yet produced sufficient evidence. Most studies have examined the feasibility of these treatment plans in GBM (18, 160, 161), with the majority of the literature focusing on animal studies in this area (162–164). Safdie et al. (165) demonstrated that fasting for 48 hours before radiotherapy or chemotherapy could sensitize GBM cell lines from mice, rats, and humans to the therapy. Furthermore, in live organisms, this fasting regimen resulted in improved survival and a significant decrease in circulating glucose and IGF1 levels. Another study by Duffy et al. (166) interestingly demonstrated that fasting selectively enhanced the cytotoxicity of TMZ in human GBM cell lines, while having no effect on normal astroglial cells. Moreover, Schreck et al. (160) investigated a fasting schedule for astrocytoma patients with grade II to IV. In this regimen, patients followed the glioma Atkins diet and fasted for 2 days during an 8-week period. The schedule was accepted by 48% of the participants, with reasonable safety and tolerability. This regimen resulted in increased levels of β-hydroxybutyrate and acetone in the brain, and decreased levels of HbA1C and insulin. It is noteworthy that altered carbohydrate metabolism is a common and important trait of cancer, well known as the “Warburg effect.” Therefore, cancer cells preferentially utilize anaerobic glycolysis, which reduces ATP production and causes cancer cells to require excessive glucose to survive. Fasting leads to decreased systemic glucose and anabolic hormones, as well as increased ketone bodies such as β-hydroxybutyrate, which results in the regression of tumors. It seems that various types of fasting are more effective, while traditionally any type of ketogenic diet has been used for this purpose. In addition to glucose, glutamine is also considered to be an important fuel for GBM cells, powering their growth. Mukherjee et al. (162) utilized a glutamine antagonist (6-diazo-5-oxo-L-norleucine) in combination with a calorie-restricted ketogenic diet as a diet/drug strategy. They demonstrated the ability to kill GBM cells, reverse symptoms, reduce edema, hemorrhage, inflammation, and enhance survival in mice with late-stage GBM. Most of the literature focuses on ketogenic metabolic therapy as an adjuvant treatment in GBM. However, its efficacy remains to be determined due to the limited nature of these studies, with most being case reports (167, 168) or animal/*in vitro* studies. However, some studies have shown that the ketogenic diet, by increasing fatty acids and ketone bodies, can lead to aggressive tumor growth through increased utilization of fats and ketones in GBM cells, and reduced survival in mouse models (20). Therefore, applying a ketogenic diet in GBM from the cellular level to clinical practice remains very challenging, and further studies are necessary for the future.

## 5 Metformin, senescence, and aging

According to the literature, various types of stress on the body, such as oxidative, psychological, or genotoxic, DNA damage, epigenetic changes, metabolic function disorders, oncogene overexpression, genetic mutations, and mitochondrial disorders, ultimately resulting in the senescence state. Senescence is a state in which cells irreversibly enter a cell cycle arrest state. A senescent cell undergoes increased volume, secretes inflammatory factors, and contributes to the creation of the SASP. Studies have shown that the accumulation of senescent cells, which commonly occurs with aging, is associated with inflammation and chronic age-related diseases (53, 169, 170). Recent studies suggest that metformin can induce anti-aging transcriptional changes (29, 158, 171). However, it is still debatable whether this effect of metformin is also true for healthy individuals, and the exact mechanisms are unknown. The potential benefit of metformin lies in reducing mortality associated with the disease by lowering glucose levels. Also, studies have shown that metformin can reverse telomere attrition, which is the most prominent characteristic of senescent cells (29, 171–174). Moreover, some top mentioned mechanisms in literature, which have been attributed to the anti-aging efficacy of metformin, consist of intervening in nutrient sensing, DNA damage, the accumulation of ROS, telomere attrition, inflammation, cellular senescence, stem cell depletion, and autophagy. These mechanisms can then be attributed to prevent aging-related diseases (171).

## 6 Metformin, senescence, and cancer, especially GBM

As described in the previous section, one of the important anti-aging properties of metformin is its anti-senescence effect. However, when focusing on cancer studies, it is evident that metformin can exert anticancer properties through various mechanisms and traits, such as inducing cell senescence, which is one of the most important mechanisms (36, 175, 176). As a result, there is a paradoxical interpretation of the effect of metformin on cell senescence. It seems that this is related to the paradigm in which the effects of metformin could be highly tissue-specific and context-dependent. In this study, we discuss the context-dependent effects of metformin for the second time. On the other hand, metformin-induced cell senescence in cancer cells, which is considered a beneficial effect of this drug on tumor initiation, may have a detrimental impact on tumor progression. This is due to the development of SASP phenotype and subsequent immunosenescence (29, 177, 178). Studying in this field is fascinating, and the exact interrelated concept is still not entirely clear. Taken together, the prevailing observation is that most diseases, particularly cancers, are age-related and tend to occur with aging. Therefore, metformin, through its established effects on delaying aging, primarily by interfering with SASP, can reduce the incidence of age-related cancers (37, 179, 180). According to available data, a plausible explanation for the bidirectional effects of metformin on senescence could be that metformin decreases the cell senescence threshold, leading to an accelerated initiation of cell senescence in response to oncogenes (36, 176, 179). Metformin induces SASP during the early stages of tumorigenesis to restore immune surveillance, promoting immune-mediated clearance of senescent cells that may act as potential premalignant agents, as well as existing malignant lesions, and to impede the evasion of cancer cells from antitumor defenses (176). Therefore, it seems that the net effect of metformin should be a significant reduction in dysfunction and malignant cells. Some studies suggest that metformin acts as a radiosensitizer in cancer therapy, enhancing radiation-induced senescence and increasing the effectiveness of radiotherapy (181–183). However, from another perspective, studies on cancer have shown that metformin exhibits anti-SASP activity (4, 29, 184, 185). It has been reported that senescent cells have the ability to undergo genomic reprogramming, allowing them to re-enter the cell cycle and regain their stemness phenotype. Furthermore, senescent cells with aggressive SASP activity could induce immunosenescence over time, leading to immune dysfunction, particularly in relation to antitumor immune responses (186). Additionally, there is evidence indicating that the accumulation of senescent cells can lead to mTOR activation and create a cancerophil microenvironment (187, 188). Based on the literature, although metformin is not an immunosuppressive agent, it appears that in a proinflammatory context such as senescence, it could inhibit the NFKB pathway, inflammation, and most cytokines related to SASP (37). Therefore, metformin has been identified as a senostatic agent due to its anti-SASP activity, which can inhibit tumor progression. Some studies have shown that the remnant of senescent cells after chemotherapy and radiotherapy is associated with cancer relapse. Metformin, as an adjuvant and effective senotherapy drug, has been found to decrease the presence of these cells (189). This belief still remains that the bidirectional effects of metformin mentioned above warrant further preclinical and clinical studies in the future. So far, the effects of metformin have been discussed in various types of cancer, including breast (94), prostate (109), ovary (95), pancreas (78), and hepatocarcinoma (25). However, research on brain cancers is relatively scarce. After conducting a comprehensive search on PubMed based on following terms and search strategy: GBM OR glioblastoma OR “glioblastoma multiform” AND senescence OR SASP OR senesc* AND metformin OR glucophage, just was funded 2 articles. Then, when (head OR neck OR cerebral OR cranial OR brain AND tumor OR carcin* OR malignan* OR neoplasm*) were substituted for GBM, the search yielded 9 results. Among them, an important recent study worth mentioning is the work of Fuentes-Fayos et al. (4) The results of their study showed that treatment with metformin and simvastatin alone, and with a stronger effect in combination, could induce a senescent state and increase the number of senescent cells. These observations were confirmed via β-galactosidase assay, along with effective alterations in key genes related to SASP in GBM cell lines. Moreover, according to their report, this treatment resulted in telomere lengthening, which is an outcome of the senescence state, as well as phosphorylation of ERK1/ERK2 and P53, and inhibition of some oncogenic factors. This led to the blocking of proliferation capacity in GBM cell lines and a decrease in aggressive features in the most aggressive form of cancer (4). However, some other studies on head and neck squamous cell carcinoma (HNSCC) have reported that an early senescence state, numerous senescent cells, and consequently SASP lead to resistance against radio (chemo) therapy. Metformin, with a decrease in senescence state, could alleviate this resistance (185). Similarly, Skinner et al. (183) showed that metformin was a radiosensitizer in HNSCC with a disruptive P53. On the other hand, Woo et al. (190) reported that metformin inhibits malic enzyme 2, leading to increased senescence mediated by ROS in HNSCC. Curry et al. (191) demonstrated that administering a diabetic dose of metformin (2000 mg/day) for 9 days or more before surgery could increase senescence in cell lines derived from HNSCC patients. Moreover, Hu et al. (185) demonstrated that metformin trough induces cell cycle arrest *in vitro* and *in vivo*, alongside modulation of SASP via inhibition of mTOR and Stat3 pathways, and has antitumor activity in HNSCC. Taken together, it is concluded that metformin should be used as a senescence inducer along with anti-SASP activity, which together account for an important part of metformin’s antitumor efficacy and may be used as a promising therapy in these patients.

## 7 Are the antitumor effects of metformin more attributed to apoptosis or senescence?

An intriguing recent study of high quality discusses how senescent cells contribute to the aggressiveness of malignant cells in mouse and human glioblastoma (192). Senescent cells accounted for approximately 7% of tumors, and their removal was associated with improving the tumor microenvironment in the study mentioned (192). This result supports our interpretation as outlined in the previous sections. According to the literature, senescence may be a beneficial phenomenon only in the initial stage of GBM, but in the progression phase, it could actually be detrimental (4, 53, 189). Therefore, it seems that apoptosis or autophagy play a fundamental role in eventually clearing senescent cells and have a greater impact on antitumor efficacy than senescence. In line with previous findings, Guarnaccia et al. (128) have demonstrated that metformin interferes with sphingolipid metabolism, leading to the production of pro-apoptotic ceramide and ultimately facilitating apoptosis in GBM cells. Furthermore, Xiong et al. (47) concluded that metformin could impact the rise in caspase 3 activity in human GBM cells. Sesen et al. (133) demonstrated that metformin could induce apoptosis by increasing the expression of the pro-apoptotic protein Bax and decreasing the expression of the anti-apoptotic protein Bcl2 in GBM cells. Furthermore, some studies have reported that metformin enhances the sensitivity of GBM cells to Temozolomide, resulting in increased apoptosis due to DNA damage (193, 194). Other studies have also reported that metformin induces apoptosis or autophagy in animal and human GBM (49, 121, 128, 133). Hence, it appears that the antitumor effects of metformin in GBM cells can be attributed to apoptosis rather than senescence.

## 8 What influential factor determines the cellular decision between apoptosis and senescence?

The cellular decision between apoptosis and senescence is influenced by complex and diverse factors, including the type and intensity of the cancer cell’s DNA damage, the tumor microenvironment, the presence of immune cells, and the overall physiological status (195–198). Typically, studies have indicated that apoptosis occurs after more extend cellular stress than senescence. In this regard, some studies have demonstrated that the dosage of anticancer drugs is an important factor. Song et al. (199) reported that lower doses of doxorubicin induce senescence, while higher doses lead to apoptosis in breast cancer cells. Yi et al. (35) reported the same result regarding metformin in hepatoma cells. Moreover, cell type is a crucial determinant, as illustrated by the study of Curry et al. (191), which showed that metformin induces senescence in stromal cells whereas exert apoptosis in carcinoma cells in head and neck cancer samples. On the other hand, some cancer studies have highlighted the role of PTEN as a key determinant in the decision-making process of cancer cells. PTEN-deficient cells enter a state of senescence, while cells with adequate PTEN levels undergo apoptosis (196, 200, 201). Some interesting results suggest a potential role for P53 in radiation-induced senescence, as one study has shown a correlation between the two. The results of this study show that breast cancer cells with a missense mutation in the DNA-binding domain of P53 are susceptible to apoptosis rather than senescence (202), while the opposite was observed in GBM cells (203). This missense mutation resulted in escape from radiation in GBM cells. In line with this, some mechanistic studies, which identified P53 as wild type, have shown that adjuvant anticancer therapies such as metformin dramatically induce SASP. This, in turn, leads to inappropriate activation of AKT/ERK-mTORC1-4EBP1-MCL1/SURVIVIN in P53-deficient cancer samples with wild type (204). However, this result has not been supported in P53 deficient cancer cells without wild type. In GBM, temozolomide, which is the first-line therapy, has interestingly been shown to induce senescence more than apoptosis. This, in turn, has been mentioned as the cause of resistance to the therapy and tumor relapse (205–207). Taken together, it seems that more future studies should evaluate the effect of metformin as a senotherapy on GBM *in vitro* and *in vivo*, taking into consideration the type of P53.

## 9 Conclusion

In the current review, we carefully evaluated the exact mechanisms that account for the therapeutic modalities of metformin in cancer settings, with specific emphasis on GBM. Our findings encourage scientists to conduct more well-designed studies that take into account crucial factors concerning metformin and GBM research, including MGMT promoter status and GBM patients without diabetes. Several studies have illustrated that metformin could induce senescence in GBM cells. However, most other studies have stated that metformin decreases some key component of SASP and plays a role in alleviating GBM. In the current review, we conclude that metformin has anti-tumor efficacy as a senescence inducer in the initiation phase of cancer. It is worth noting that senescence, along with SASP production, is not a beneficial phenomenon in the progression phase of cancers. Metformin has been shown to act as an anti-senescent agent during this stage, as indicated by related studies. Actually, metformin has anti-SASP activity, which is a common factor explaining its anti-aging and anti-cancer effects. Moreover, the effects of metformin are highly context-dependent. As a good example of this, metformin leads to increased insulin sensitivity in active metabolic organs, while it inhibits insulin signaling in the GBM microenvironment. This effect has also been verified in other types of cancer. On the other hand, cells in which mTOR signaling is more active undergo a senescent state, while those with inhibited signaling enter a quiescent state that eventually results in apoptosis. Senescent cells, however, are resistant to apoptosis. Studies have indicated that the primary anti-tumor efficacy of metformin is associated with apoptosis rather than senescence. The key determinants for cancer cells to choose between apoptosis and senescence include cell types, PTEN, P53 type and its mutations, and the mTOR activity of cells. Finally, although the vast mechanisms have been discussed and metformin effects have often been reported as effective, some clinical trials have shown that metformin has no effect on clinical outcomes such as survival and tumor recurrence. Therefore, it seems that there is still a need for studies with a more meticulous perspective on related mechanisms to clarify existing controversies, especially in cases of GBM. In the next step, it is recommended that more extensive and rigorous clinical trials be conducted to either approve or reject the clinical efficacy of metformin as a senotherapeutic agent in the GBM treatment protocol.
